# Significant Hair Regrowth With 5% Topical Minoxidil in a Child With Marie Unna Hereditary Hypotrichosis Caused by a Recurrent *HRURF* Variant

**DOI:** 10.1111/jocd.70382

**Published:** 2025-08-12

**Authors:** Can Cui, Xi Chen, Ying‐Zi Zhang, Jin‐Yuan Ma, Ai‐Hua Wei

**Affiliations:** ^1^ Department of Dermatology Beijing Tongren Hospital, Capital Medical University Beijing China

To the Editor,

## 1 Introduction

Marie Unna hereditary hypotrichosis (MUHH) is a rare autosomal dominant disorder first described in 1925 [1]. Affected individuals typically present at birth with sparse or absent scalp hair, eyebrows, eyelashes, and body hair. In 2009, *HRURF* was identified as the causative gene for MUHH1 [2]. Currently, no targeted therapy is available.

## 2 Case Report

A 4‐year‐old girl presented with congenital hypotrichosis. At birth, her scalp hair was sparse and shed within the first 3 months. Hair regrowth was minimal, never exceeding 5 cm in length. She was born at term via spontaneous vaginal delivery with normal development. Her father had androgenetic alopecia, and her mother had a normal hair phenotype. There was no consanguinity or family history of similar conditions.

**FIGURE 1:**
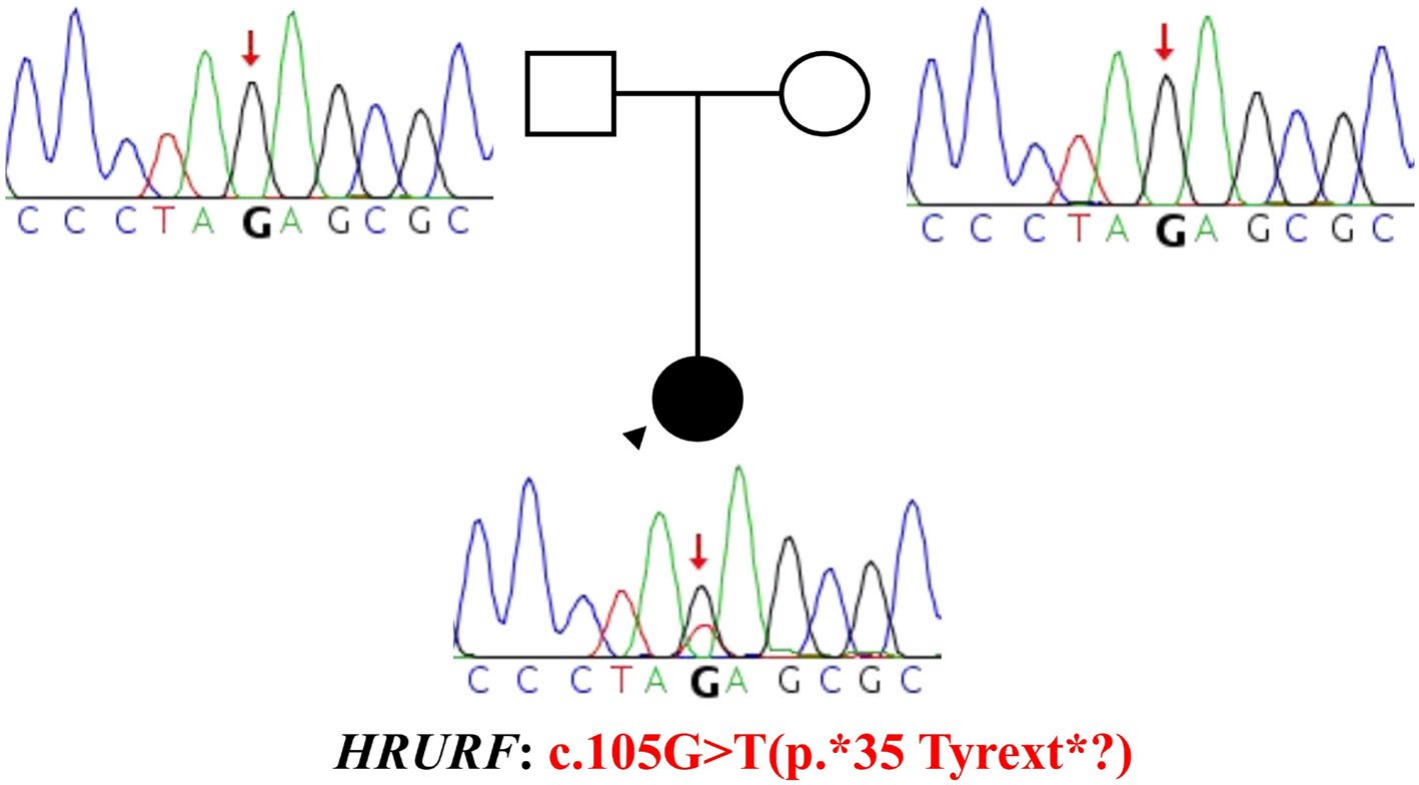
Genetic analysis of the *HRURF* variant: Sanger sequencing confirmed a de novo *HRURF* stop‐loss variant (c.105G > T, p.*35 Tyrext*?) identified by whole‐exome sequencing.

Physical examination revealed diffuse, fine, slightly curled scalp hair with androgenetic distribution. Eyelashes were thin and curled, and eyebrows were nearly absent. A widened midline diastema was noted. No abnormalities were observed in skin, nails, or other systems. After obtaining written informed consent, peripheral blood samples were collected from the patient and both parents. Whole‐exome sequencing identified a de novo stop‐loss variant in *HRURF* (c.105G > T, p.*35Tyrext*?), confirmed by Sanger sequencing. Both parents were wild‐type. The patient was diagnosed with MUHH1 (Figure 1).

**FIGURE 2:**
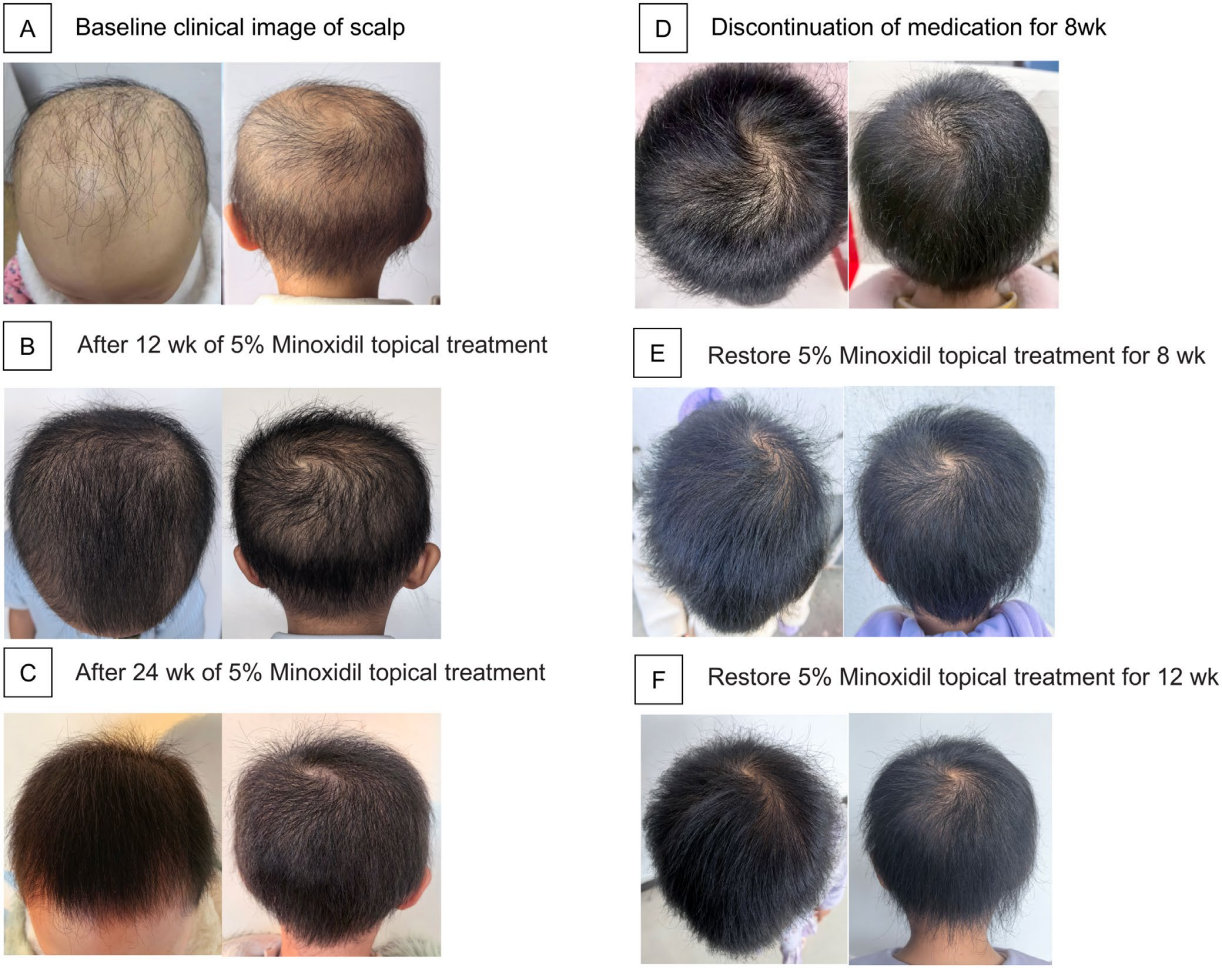
Clinical progression of scalp hair growth and response to 5% Minoxidil topical treatment. (A) Baseline clinical image of scalp before treatment. (B) Scalp appearance after 12 weeks of 5% Minoxidil application. (C) Scalp appearance after 24 weeks of 5% Minoxidil application. (D) Scalp appearance following 8 weeks of discontinuation of medication. (E) Scalp recovery after restarting 5% Minoxidil treatment for 8 weeks. (F) Scalp improvement after resuming 5% Minoxidil treatment for 12 weeks.

Given strong parental interest, topical 5% minoxidil was initiated once daily to the scalp (solution for 4 weeks). The parents later switched to the foam formulation without medical instruction, and no adverse effects were reported. After 12 weeks, significant scalp hair regrowth was observed, with improved density and no reported irritation or hypertrichosis. At 6 months, scalp hair density further improved. Treatment was discontinued for 8 weeks, during which minimal hair breakage was noted. Hair length increased slightly, but density plateaued. Upon observing breakage, treatment was resumed. After 8 weeks of re‐initiation, hair density increased, and by 12 weeks, hair length exceeded 10 cm (Figure 2).

## 3 Discussion

Over 25 pathogenic variants in *HRURF* have been identified in MUHH1 [3]. We identified a recurrent stop‐loss variant in *HRURF* (c.105G > T, p.*35Tyrext*?), previously reported in a Danish family, which may convert the normal stop codon into a tyrosine codon, potentially extending the open reading frame and elongating the HRURF protein [3]. In the Danish family, the mother carrying the same variant had sparse, curly, and frizzy hair at birth, but her hair density and appearance normalized in adulthood. However, her two children with the same variant presented with congenital hypotrichosis. In our case, the patient showed significant hair regrowth after topical minoxidil treatment. Whether this improvement will persist or resemble the natural course observed in the Danish mother requires further follow‐up. Nevertheless, the marked short‐term efficacy supports the potential benefit of topical minoxidil in such cases.

Evidence for treating congenital hypotrichosis remains limited. Minoxidil promotes anagen entry and prolongs follicular activity, enhancing hair density and diameter. Topical 5% minoxidil has been used in children as young as 2 years, though hypertrichosis is a known side effect [4]. Case reports have demonstrated its potential to improve hair density in monilethrix patients with *KRT86* mutations without notable adverse effects [5]. In a recent case report, an 8‐year‐old boy with CDSN‐related hypotrichosis simplex of the scalp (HSS) received a combination of topical 5% minoxidil and botanical extracts. After 4 months of treatment, marked clinical improvement was observed, with a reported 70% increase in hair density at 6‐month follow‐up [6]. While long‐term safety remains uncertain, minoxidil may serve as a symptomatic option in selected pediatric patients under close supervision.

## 4 Conclusion

This case demonstrates that 5% topical minoxidil may be effective in improving hair density in patients with MUHH. The clinical response suggests potential benefit even in congenital, genetically driven hair loss. Combined with previous reports on monilethrix and hypotrichosis simplex, our findings support expanding the use of minoxidil in rare hereditary alopecias. Early intervention and individualized treatment approaches may offer improved outcomes for these otherwise treatment‐refractory conditions.

## Author Contributions

Ai‐Hua Wei conceived and supervised the study; Jin‐Yuan Ma, Ying‐Zi Zhang, and Can Cui analyzed data; Can Cui wrote the manuscript; Xi Chen made manuscript revisions. All authors reviewed the results and approved the final version of the manuscript.

## Consent

Written patient consent for publication was obtained.

## Conflicts of Interest

The authors declare no conflicts of interest.

## Data Availability

The authors confirm that the data supporting the findings of this study are available within the article.
